# Urotropine in Typhoid Fever

**Published:** 1900-04-28

**Authors:** 


### UROTROPINE IN TYPHOID FEVER.

The most interesting point made by Dr. Horton-Smith in his Goulstonian lectures in regard to the treatment of typhoid fever is what he says about the effect of urotropine in destroying the typhoid bacilli, so often contained in the urine of patients suffering from that disease. The action of this drug may be regarded as specific in regard to typhoid bacilluria and cystitis. Urotropine is formed by the action of formalin on ammonia, and was introduced six years ago into medical practice by Dr. Arthur Nicolaier as a urinary antiseptic. It was soon found to have considerable value in checking the decomposition of the urine in cases of ammoniacal cystitis, the effect being due, as Dr. Nicolaier has shown, to the liberation of free formalin from the drug after its passage into the urine. It is to be noted, however, that in the acid cystitis produced by the bacillus coli it is almost without effect, so that it cannot be regarded, as some have too hastily thought, as a specific for all forms of urinary infection. So far, however, as concerns typhoidal affections of the urinary tract, it fully deserves this name. Urotropine may be given in doses of 10 grains three times a day, and it seems to take effect almost at once. "So marked and immediate, indeed, is the effect of the drug, that if the urine is not clear at the end of 24 hours we may at once suspect most strongly that the bacillus present is not the typhoid bacillus, but one of the varieties of the bacillus coli." Sometimes a permanent cure may be effected by the time 60 grains have been taken, but to make certain it would be well in all cases to continue the drug for at least a week. It seems clear then that urotropine should be used whenever a urinary complication due to the typhoid bacillus has occurred. But seeing that the presence of the bacilli in the urine is always a source of danger to others, even when no symptoms are complained of, the question arises whether this drug should not be given to the extent of 30 grains daily in all cases of typhoid fever throughout its whole course and during the first three weeks of convalescence. It produces no ill effect beyond occasionally some urethral pain if the urine is allowed to become concentrated, and there can be no doubt that in many cases, especially in poor houses, where infection from cases is unfortunately far too common, much of this risk of so-called sick-room infection would be done away with if the urine could be rendered innocuous in this respect. It is to be remembered that typhoidal bacilluria, which presumably is infectious, often continues for a long time after the fever has subsided, and that it is worth a good deal to be able in such a simple way to put a stop to so insidious a source of infection.

